# Lifetime and 12-month prevalence of eating disorders amongst women in mid-life: a population-based study of diagnoses and risk factors

**DOI:** 10.1186/s12916-016-0766-4

**Published:** 2017-01-17

**Authors:** Nadia Micali, Maria G. Martini, Jennifer J. Thomas, Kamryn T. Eddy, Radha Kothari, Ellie Russell, Cynthia M. Bulik, Janet Treasure

**Affiliations:** 1 Eating and Weight Disorders Program, Department of Psychiatry, Icahn School of Medicine at Mount Sinai, New York, NY USA; 2 Mindich Child Health and Development Institute, Icahn School of Medicine at Mount Sinai, New York, NY USA; 3 Institute of Child Health, University College London, London, UK; 4 Department of Psychiatry, Harvard Medical School, Boston, MA USA; 5 Eating Disorders Clinical and Research Program, Massachusetts General Hospital, Boston, MA USA; 6 Department of Psychological Medicine, Cardiff University, Cardiff, UK; 7 Department of Psychiatry, University of North Carolina at Chapel Hill, Chapel Hill, NC USA; 8 Department of Medical Epidemiology and Biostatistics, Karolinska Institutet, Stockholm, Sweden; 9 Department of Nutrition, University of North Carolina at Chapel Hill, Chapel Hill, NC USA; 10 Eating Disorders Research Unit, Psychological Medicine, Institute of Psychiatry, King’s College London, London, UK

**Keywords:** Eating disorders, Anorexia nervosa, Bulimia nervosa, Binge eating disorder, ALSPAC, Women, Mid-life, Prevalence, Risk factors, Childhood

## Abstract

**Background:**

Eating disorders (EDs) are common amongst women; however, no research has specifically investigated the lifetime/12-month prevalence of eating disorders amongst women in mid-life (i.e., fourth and fifth decade of life) and the relevant longitudinal risk factors. We aimed to investigate the lifetime and 12-month prevalence of EDs and lifetime health service use and to identify childhood, parenting, and personality risk factors.

**Methods:**

This is a two-phase prevalence study, nested within an existing longitudinal community-based sample of women in mid-life. A total of 5658 women from the UK Avon Longitudinal Study of Parents and Children (ALSPAC; enrolled 20 years earlier) participated. ED diagnoses were obtained using validated structured interviews. Weighted analyses were carried out accounting for the two-phase methodology to obtain prevalence figures and to carry out risk factor regression analyses.

**Results:**

By mid-life, 15.3% (95% confidence intervals, 13.5–17.4%) of women had met criteria for a lifetime ED. The 12-month prevalence of EDs was 3.6%. Childhood sexual abuse was prospectively associated with all binge/purge type disorders and an external locus of control was associated with binge-eating disorder. Better maternal care was protective for bulimia nervosa. Childhood life events and interpersonal sensitivity were associated with all EDs.

**Conclusions:**

By mid-life a significant proportion of women will experience an ED, and few women accessed healthcare. Active EDs are common in mid-life, both due to new onset and chronic disorders. Increased awareness of the full spectrum of EDs in this stage of life and adequate service provision is important. This is the first study to investigate childhood and personality risk factors for full threshold and sub-threshold EDs and to identify common predictors for full and sub-threshold EDs. Further research should clarify the role of preventable risk factors on both full and sub-threshold EDs.

**Electronic supplementary material:**

The online version of this article (doi:10.1186/s12916-016-0766-4) contains supplementary material, which is available to authorized users.

## Background

Eating disorders (EDs) are severe psychiatric disorders associated with high levels of morbidity [1], mortality [2, 3], and social, psychological and physical impairment [4]. The Diagnostic and Statistical Manual for Mental Disorders 5^th^ edition (DSM-5) [5] recently broadened ED diagnostic criteria, aiming to reduce the number of individuals with an ED who do not fit full-threshold diagnostic categories. Binge-eating disorder (BED) was introduced as a diagnostic category, and the criteria for anorexia nervosa (AN) and bulimia nervosa (BN) were broadened. Although previously considered low prevalence disorders, this broadening of the diagnostic criteria in DSM-5 has yielded preliminary evidence that ED are more common than once thought. A small number of community-based studies have investigated the prevalence of DSM-5 EDs. Lifetime prevalence estimates of DSM-5 EDs have varied dramatically across studies [6, 7] and the same applies to period-prevalence estimates [8, 9]. The remarkable variability across studies is likely due to sample size, differences in study design (questionnaire only assessment vs. two-phase studies), and a focus on adolescents/young adults only (reflecting the peak age of onset of AN and BN), thus highlighting a need for further large studies. No previous studies have investigated the period or lifetime prevalence of EDs amongst women in the fourth and fifth decade of life, after most individuals would be considered to have passed through the primary window of risk. We recently highlighted a wide gap in access to healthcare amongst adults with ED in a UK population-based sample [8], and we therefore sought to replicate and extend our findings.

A long-term perspective offers the unique opportunity to investigate EDs and relevant precursors/risk factors using a disease life-course approach. Few studies have investigated risk factors for EDs using a longitudinal prospective design [10], and the majority have focused on treatment seeking samples and full threshold EDs. Prior evidence from our group [11, 12] and others [13, 14] points to childhood experiences and personality as important risk factors for EDs; however, there is a relative lack of population-based studies investigating these in relation to ED. Thus, our aims were (1) to determine the lifetime and 12-month prevalence of DSM-5 EDs in mid-life in women from a population-based cohort using a two-phase design and to explore healthcare access, as well as (2) to investigate associations between lifetime ED, risk factors (personality characteristics (personality, locus of control); early childhood experiences (sexual abuse, maternal care, carer/parent death, parental separation/divorce and being under local authority care)) and fixed factors (intelligence quotient (IQ)).

## Methods

### Sample

The Avon Longitudinal Study of Parents and Children (ALSPAC) is a population-based, extensive prospective study of women and their children, investigating the effects of environment, genetic and other factors on child health and development [15]. All pregnant women living in the geographical area of Avon, UK, who were expected to deliver their baby between April 1, 1991, and December 31, 1992, were invited to take part in the study. Uptake was high and those enrolled represented approximately 85% of the eligible population. ALSPAC recruited 14,541 pregnant women; all women gave informed and written consent. The study website contains details of all the data that is available through a fully searchable data dictionary: http://www.bris.ac.uk/alspac/researchers/data-access/data-dictionary/.

### Procedures and measures

**Fig. 1:**
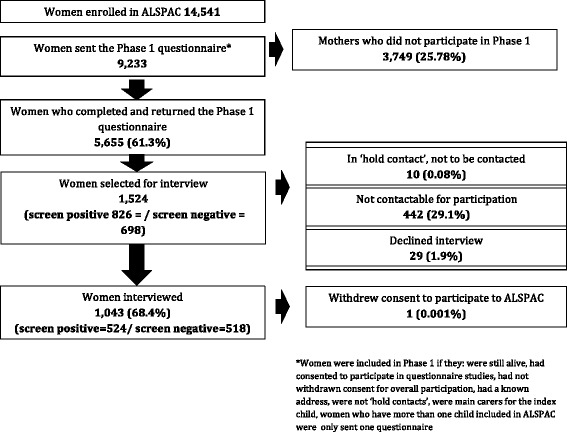
Flowchart describing study participation

Data collection was carried out in two phases between 2009 and 2012. Figure 1 presents the study participation flowchart.

#### Phase 1

A total of 9233 women who were still alive, enrolled in the study and participating in assessment waves, and were main carers for their ALSPAC child, were enrolled and sent a version of the Eating Disorders Diagnostic Schedule (EDDS) adapted to cover the whole lifespan [16]. Women were invited to complete the questionnaire either online or on paper. Screen positive and a similar percentage of screen negative (~10%) women were selected for interview, based on sample size calculations. Criteria for screening positive were based on a previous study and identified diagnostic cut-offs [16].

#### Phase 2

Women who screened positive and a subset of those who screened negative were interviewed using the ED section of the Structured Clinical Interview for DSM-IV-TR disorders (SCID-I) (with no skip rules) [17], supplemented with a version of the LIFE interview [18], adapted to EDs [19], aimed at investigating presence, frequency, and duration of ED behaviors (restriction, fasting, excessive exercise, binge eating, and purging), as well as body mass index (BMI) over the lifetime. Women were asked to anchor their responses using major life events, such as the birth of their study child, in order to increase accuracy of reporting and minimize reporting bias. Each ED behavior was recorded over the lifetime from its first occurrence to time of the interview. Diagnoses were obtained supplementing the information for the SCID-I with detailed information from the LIFE to obtain DSM-5 diagnoses for disorders (e.g., BN and BED) reflecting different frequency thresholds in DSM-IV and DSM-5. Questions about access to healthcare and treatment for ED were specifically devised for the purpose of this study; if women reported any ED behaviors or cognitions they were asked if they had ever sought and/or received treatment for these. If they replied yes they were then asked to describe what kind of treatment they had received (inpatient, outpatient, psychological treatment, medication or other); they were also asked if they had received treatment for other psychiatric disorders.

### Training and quality control

Interviews were carried out by three trained interviewers (all psychologists). All interviewers practiced the interview amongst themselves and with colleagues, and conducted interviews under supervision prior to interviewing study subjects, including rating available interviews. Interviewers attended a monthly meeting with the first author, where interviews of symptomatic individuals were discussed. All diagnoses were reviewed and confirmed by the first author. A subset of interviews were recorded for inter-rater reliability purposes. Interviewers demonstrated excellent inter-rater reliability on the SCID with 100% agreement; the intraclass correlation coefficient for diagnosis was 1.00.

### Diagnostic properties of the EDDS

The adapted EDDS had a sensitivity of 97.3% (95% CI, 94.9–98.8%) and specificity of 74.6% (71.1–77.8%), and positive and negative predictive values of 65.1% and 98.3%, respectively. False negatives were therefore rare, whilst false positives were more common.

### ED diagnoses

Diagnoses of DSM-5 ED (AN, BN, BED, sub-threshold BN and BED, purging disorder (PD), and other specified feeding and eating disorder (OSFED)) were obtained using the SCID supplemented with behavioral data (including frequency and duration of each symptom) from the LIFE. ED diagnoses were derived as shown in Additional file 1: Table S1. Given the age of our sample and the diagnostic instruments used, we were unable to ascertain the prevalence of avoidant/restrictive food intake disorder, pica, or rumination.

### Risk factors

Data on relevant predictors were obtained as part of routine ALSPAC data collections approximately 20 years prior to the current study.

### Obtained during pregnancy (12, 18, 32 weeks gestation)

#### Childhood unhappiness

Ascertained using women’s rating of their happiness in childhood (up to 16 years). We derived a binary variable (very unhappy, quite unhappy, and not really happy vs. moderately happy, very happy).

#### Parental divorce or separation, adoption or being under health authority care, death of a carer

Assessed by asking women whether their parents had divorced or separated prior to their 18th birthday; whether they had been legally adopted or had been placed under local authority care (foster care and or group homes); and whether a parent or person who cared for them had died prior to their 17th birthday. These variables were retained as dichotomous (yes or no).

#### Early sexual abuse

Assessed using a questionnaire about early sexual experiences covering a range of sexual experiences (including noncontact exposure, fondling, oral sex, and sexual intercourse) involving boy/girlfriends, parents, other relatives, family friends, and strangers. Experiences involving physical sexual contact with an individual other than a boy/girlfriend prior to age 16 were defined as sexual abuse. A dichotomous variable (childhood sexual abuse vs. none) was generated.

#### Life events

Data on life events experienced up to age 17 were obtained from a questionnaire completed during pregnancy, containing a life-event inventory (indicating occurrence of the event), with five response categories for each event (indicating the extent to which the respondent was affected) based on Brown & Harris’s work [20, 21]. In order to take into account both the wide variation of life events severity and their impact ratings, life events were weighted to create a continuous score as previously reported [22].

#### Bonding with parents

Assessed using the Parental Bonding Instrument [23]. Two scores were derived, (1) parental over-protection (degree to which women felt that their own parents had been over-protective and failed to allow them to make their choices in childhood – higher scores indicate a more oppressive relationship) and (2) maternal care (measured the woman’s perception of the relationship she had with her own mother – higher scores indicate a warmer relationship). We categorized the latter according to the top and bottom quartile, with the interquartile scores being the referent, to determine whether a warm relationship (top quartile) would be protective and a poor relationship (bottom quartile) would be risk-conferring for EDs.

#### Locus of control (LOC)

Assessed with a shortened version of the Adult Nowicki-Strickland Internal/External Locus of Control Scale [24], measuring external (higher scores) versus internal LOC.

#### Interpersonal sensitivity

Measured using the Interpersonal Sensitivity Measure [25] a valid and reliable measure assessing sensitivity to interpersonal and social feedback, and interpersonal avoidance [26].

### Fixed factor

#### General intelligence

Measured using the Wechsler Abbreviated Scale of Intelligence [27] 15–16 years after enrolment in the study on 2165 women. We used the total IQ score.

### Covariates

Maternal age was obtained during Phase 1; women’s ethnicity and educational status were obtained combining data provided at various time-points between enrolment and child age 18 [28].

### Statistical analyses

All analyses were carried out using STATA 13 [29].

### Prevalence

Prevalence estimates were calculated allowing for the two-phase sampling procedure by using weights [30, 31]. A sampling weight was generated using information from Phase 1 and the Phase 2 sampling strategy as described by Dunn et al. [29]. The sampling weight indicates how many Phase 1 participants each participant in Phase 2 represents. The weighted prevalence estimates of ED diagnoses from diagnostic interviews in Phase 2 were weighted back to the sample that participated to Phase 1. The survey (svy) set of commands was used in STATA to obtain prevalence estimates and carry out regression analyses, as they allow for stratified sampling and provide robust estimation of 95% confidence intervals (CIs).

### Risk factors analyses

Analyses were adjusted for the a priori confounders of maternal age, ethnicity and education. Given high diagnostic crossover [32], women were categorized into mutually exclusive diagnostic groups. Women who only met one diagnosis were assigned to that diagnostic group; for those who had more than one diagnosis over their lifetime a hierarchical approach was used: full diagnoses (AN, BN, BED) trumped OSFED subtypes, BED trumped BN, and BN trumped AN, in accordance to our and others’ previous studies [19, 33], and evidence that diagnostic crossover over the lifetime in ED and in this sample occurs most commonly from restrictive type disorders (anorexia nervosa – restrictive (AN-R)) to binge and/or binge-purge disorders [19, 32].

Sensitivity analyses were carried out by removing from the analytic sample women who met criteria for more than one ED and assessing differences in association estimates with hypothesized risk factors. Women with complete data on exposures and outcomes were included in these analyses. Missing data on covariates (1%) were imputed using multiple imputation by chained equation performed in STATA 13. Imputation models included all variables in the analyses and predictors of missingness. Results obtained after imputation were similar to those found using complete case analyses; therefore, we report results from multiple imputation.

All tests were two tailed and a *P* value of 0.05 was used as a cut-off for statistical significance.

## Results

### Phase 1

Amongst women who were sent a questionnaire, 5655 (61.3%) returned completed questionnaires and 826 (14.88%) were screen positive. Amongst 4832 screen negative women, 698 (12%) were randomly selected for interview in Phase 2. Characteristics of participants in Phase 1 are shown in Additional file 1: Table S2; women who participated were more likely to have received secondary education and less likely to have had prior pregnancies. The prevalence of self-reported ED at enrolment (in pregnancy) did not differ. The average age of women who participated in the study was 47.78 years (SD: 4.5).

### Phase 2

Amongst the 1524 women selected for interview, 1043 (68.4%) consented to participate in Phase 2 and were interviewed. Of those not interviewed, 10 were, at the time of interview, considered ineligible for participation by the ALSPAC study team (i.e., experiencing major life difficulties of a kind that made participation not possible such as bereavement and severe physical illness in the family), 29 (1.9%) declined participation and 442 (29.1%) were not contactable. One woman withdrew consent for participation in ALSPAC following interview, she was therefore excluded from all analyses. Interview data were therefore available for 1042 women.

Women who were interviewed within each stratum (screen positive or negative) did not differ on socio-demographic (parity, pre-pregnancy BMI, age, education) and screening (weight and shape concern, binge-eating, compensatory behaviors) characteristics from those not interviewed.

### Prevalence of ED

Table 1 presents the lifetime and 12-month weighted prevalence of ED. The weighted lifetime prevalence of ED was 15.33% (95% CI, 13.48–17.42%) and the 12-month prevalence was 3.61% (3.00–4.35%). Amongst full threshold disorders, DSM-5 AN was the most common lifetime ED (3.64%). OSFED was highly prevalent, affecting 7.64% of women in their lifetime. EDs were common in the 12 months prior to assessment (weighted prevalence: 3.61%); BED was the most common full-threshold disorder (1.03%). New onset EDs represented 41.6% of 12-month prevalent diagnoses.

**Table 1:** Weighted lifetime and 12-month prevalence of eating disorders amongst 5542 participants

	N	Weighted lifetime prevalence, % (95% CI)	N	Weighted 12-month prevalence, % (95% CI)
Any eating disorder	332	15.33 (13.48–17.42)	108	3.61 (3.00–4.35)
Anorexia nervosa (all)	105	3.64 (2.81–4.72)	7	0.23 (0.16–0.47)
Anorexia nervosa restrictive	51	2.05 (1.40–3.01)		
Anorexia nervosa binge-purge	54	1.68 (1.28–2.21)		
Bulimia nervosa	68	2.15 (1.70–2.74)	14	0.41 (0.24–0.70)
Binge eating disorder	62	1.96 (1.52–2.51)	33	1.03 (0.73–1.46)
OSFED (all)	211	7.64 (6.32–9.24)	56	1.65 (1.26–2.17)
Purging disorder	36	1.28 (0.85–1.92)	7	0.23 (0.11–0.47)
Sub-threshold bulimia nervosa	46	1.42 (1.06–1.90)	14	0.44 (0.27–0.74)
Sub-threshold binge eating disorder	30	0.90 (0.63–1.30)	13	0.38 (0.22–0.67)
Atypical anorexia nervosa	51	1.70 (1.22–2.39)	12	0.35 (0.20–0.63)
Other OSFED	49	2.14 (1.43–3.22)	10	0.29 (0.16–0.55)

Note: Two women (0.09% (0.03–0.29)) were diagnosed as unspecified feeding or eating disorder

*OSFED* other specified feeding and eating disorder

Median age of onset for the first ED diagnosis was lowest for AN-R (16, range 11–39) and highest for sub-threshold BED (26, range 13–44). The majority of women (76.3%) reported the onset of their ED to be prior to the birth of the index child. Only 27.4% of all women with EDs had sought help or received treatment for an ED at any point in their life. The most common healthcare service use was having seen a general practitioner (8.2%); 4 (1.2%) women reported having seen a psychiatrist for their ED and 4 (1.2%) having received inpatient treatment; 16 (4.9%) women reported having received individual psychological treatment for their ED; and 13 (4.0%) reported having received psychological treatment for another disorder.

### Risk factors

Amongst early risk factors, differences emerged across EDs. Having experienced death of a carer was associated with seven-fold increased odds for PD. Parental separation or divorce in childhood was associated with increased odds for BN, BED, and atypical AN. Child sexual abuse was associated with all disorders with binge-eating behaviors (anorexia nervosa binge-purge (AN-BP), BN, BED, and sub-threshold BN and BED) (Table 2). Sexual abuse perpetrated by a non-stranger was two-fold more prevalent amongst women with AN-BP, but as prevalent as sexual abuse by a stranger for BN and BED.

**Table 2:** Adjusted ^a^associations between eating disorders, ^b^correlates and precursors amongst 5320 women: OR (95% CI) from multivariable logistic regression and mean differences (95% CI) from weighted multivariable linear regression

	AN-R (N = 30)	AN-BP (N = 41)	BN (N = 55)	BED (N = 61)	Sub-threshold BN (N = 21)	Sub-threshold BED (N = 16)	Atypical AN (N = 28)	Other OSFED (N = 18)	PD (N = 27)
Risk factors	OR (95% CI)	OR (95% CI)	OR (95% CI)	OR (95% CI)	OR (95% CI)	OR (95% CI)	OR (95% CI)	OR (95% CI)	OR (95% CI)
Adopted or taken into care	(N = 27)	(N = 40)		(N = 59)	(N = 19)		(N = 27)		(N = 26)
	–	2.35 (0.59–9.28)	0.44 (0.06–3.39)	1.43 (0.40–5.19)	–	1.27 (0.16–9.88)	0.79 (0.09–7.19)	–	0.73 (0.01–6.01)
Death of carer		(N = 42)							
	0.83(0.22–3.16)	0.99 (0.28–3.48)	1.16 (0.44–3.10)	1.72 (0.71–4.13)	1.31 (0.26–6.69)	0.59 (0.1–5.48)	2.27 (0.68–7.59)	2.27 (0.62–8.32)	7.12** (2.32–21.85)
Parental separation or divorce		(N = 40)			(N = 20)				
	2.51(0.83–7.58)	1.06 (0.42–2.66)	2.02* (1.10–3.75)	2.01* (1.06–3.83)	–	1.45 (0.41–5.07)	2.49* (1.02–6.05)	0.25 (0.03–2.05)	0.85 (0.31–2.25)
Child sexual abuse (any)	(N = 28)		(N = 54)	(N = 59)			(N = 27)	(N = 17)	(N = 25)
	1.83 (0.72–4.65)	3.81*** (1.95–7.43)	4.70*** (2.60–8.50)	3.42*** (1.95–5.99)	3.23* (1.30–8.00)	8.11*** (2.74–23.94)	2.16 (0.90–5.15)	3.11 (0.77–12.60)	1.88 (0.79–4.48)
Childhood unhappiness			(N = 54)						
	2.52*** (1.19–5.34)	1.95 (0.87–4.38)	4.58*** (2.56–8.20)	3.66*** (2.01–6.68)	1.61 (0.50–5.16)	1.91 (0.52–6.96)	1.92 (0.70–5.25)	2.97 (0.98–8.99)	2.65* (1.17–6.00)
Weighted life event score	(N = 29)	(N = 42)		(N = 60)	(N = 22)				(N = 26)
	1.05* (1.01–1.09)	1.06*** (1.03–1.08)	1.08*** (1.05–1.11)	1.08*** (1.05–1.11)	1.04* (1.00–1.07)	1.06** (1.02–1.11)	1.10*** (1.06–1.14)	1.01 (0.95–1.08)	1.04* (1.00–1.08)
Parental bonding									
Maternal care		(N = 42)	(N = 55)						
Top quartile	0.75 (0.52–1.09)	0.84 (0.70–1.02)	0.79* (0.67–0.94)	0.90 (0.77–1.05)	0.76 (0.56–1.04)	0.85 (0.60–1.19)	1.09 (0.88–1.35)	1.17 (0.83–1.64)	0.94 (0.72–1.24)
Bottom quartile	1.83 (0.87–3.82)	1.62 (0.75–3.51)	2.30* (1.26–4.20)	1.95* (1.03–3.68)	0.99 (0.28–3.6111)	6.73*** (2.32–19.54)	1.72 (0.63–4.65)	1.69 (0.53–5.41)	3.40* (1.52–7.58)
Parental overprotection	1.01 (0.94–1.10)	1.09* (1.02–1.16)	1.08* (1.01–1.16)	1.13** (1.05–1.21)	1.13** (1.05–1.23)	1.02 (0.86–1.21)	1.12* (1.02–1.23)	1.10 (0.93–1.31)	1.19*** (1.10–1.29)
Locus of control	1.05 (0.92–1.20)	1.17 (0.99–1.38)	1.05 (0.93–1.20)	1.19* (1.01–1.39)	1.20 (0.89–1.62)	1.10 (0.85–1.43)	1.15 (0.92–1.43)	1.11 (0.85–1.43)	1.11 (0.93–1.33)
Interpersonal sensitivity			(N = 56)						(N = 26)
	1.05* (1.02–1.08)	1.05*** (1.03–1.07)	1.06*** (1.04–1.08)	1.04*** (1.02–1.06)	1.03** (1.01–1.05)	1.04* (1.01–1.07)	1.04** (1.02–1.07)	0.98 (0.94–1.02)	1.02 (0.99–1.04)
Fixed factors									
WASI total IQ score^c^	1.01 (0.97–1.05)	1.04* (1.01–1.07)	1.02 (0.99–1.05)	1.02 (0.98–1.06)	1.02 (0.97–1.07)	1.04 (0.97–1.10)	1.01 (0.95–1.07)	0.97 (0.93–1.01)	1.02 (0.97–1.06)

*P ≤ 0.05; **P ≤ 0.001; ***P < 0.0001; 4522 women are the referent group

^a^All analyses are adjusted for ethnicity, age at assessment, educational status

^b^N of women in each diagnostic group are shown in each cell if they differ from the overall N indicated in the top row

^c^Available on n = 2165, adjusted for ethnicity and age at assessment

*AN-R* anorexia nervosa-restrictive, *AN-BP* anorexia nervosa-binge-purge, *BN* bulimia nervosa, *BED* binge eating disorder, *OSFED* other specified feeding and eating disorder, *PD* purging disorder, WASI Wechsler Abbreviated Scale of Intelligence

Childhood unhappiness was associated with higher odds of AN-R, BN, BED, and PD. Childhood life events were positively associated with all ED (apart from other OSFED), with a 4–10% increased odds per unit score increase (Table 2). Reporting low maternal warmth (lowest quartile) was also associated with increased odds for BN, BED, and sub-threshold BED and PD. In contrast, women reporting high maternal warmth (top quartile) had 20% decreased odds of developing BN compared to those in the lowest 75% range. Women who reported a more oppressive relationship with parents had higher odds of AN-BP, BED, sub-threshold BN, atypical AN, and PD (Table 2). Amongst personality characteristics, a more external LOC was positively associated with BED, with a 19% increase in odds per one-point score increase. Higher levels of interpersonal sensitivity were positively associated with all EDs (apart from other OSFED and PD) (Table 2).

A marginal association was identified between higher total IQ and lifetime AN-BP, with a one-point increase in total IQ increasing the odds of AN-BP by 4% (OR = 1.04, 95% CI, 1.01–1.07).

Sensitivity analyses showed that, when analyses were restricted to women who had not transitioned to a different ED, the identified associations with risk factors did not change in magnitude or significance apart from the associations between maternal care and BN and BED becoming smaller and non-significant. Sensitivity analyses stratifying according to whether the ED disorder had onset before birth of the index child or after showed no differences in magnitude of associations apart from a smaller association between life events and AN-BP.

## Discussion

In this large sample of UK women in mid-life DSM-5 EDs were common. The lifetime prevalence of DSM-5 AN was higher than previously reported for DSM-IV AN but comparable to prior estimates of ‘broad’ DSM-IV AN [34] and expected given the removal of the amenorrhea criterion in DSM-5 [34], and the older age of our sample. The lifetime prevalence of BN and BED were also in line with previous community studies [35], although surprisingly, BED was less common during lifetime compared to AN and BN. This might be due to a higher percentage of highly educated women participating in Phase 1, and the ethnic composition of ALSPAC [15]. EDs other than AN, BN, and BED, now subsumed under OSFED, were common in this sample (7.6%), in particular the residual unspecified category (other OSFED). This suggests that, despite efforts in DSM-5 to reduce the prevalence of the ‘unspecified’ category (a goal of the revisions to DSM-IV), as previously shown [4, 6, 9], many individuals in the community experience EDs other than AN, BN, and BED. The relatively large subset of women presenting with ‘other OSFED’ (27.6% of all OSFED) is in line with our own [4] and others’ research [36].

EDs in the year prior to interview were more common than expected, no previous study – to our knowledge – has investigated the period prevalence of DSM-5 ED in a community sample in mid-life. OSFED was the most common ED, accounting for almost half of all prevalent ED cases and BED was the most common full-threshold disorder. These findings highlight, for the first time, that EDs are not confined to earlier decades of life and that both chronic and new onset disorders are apparent in this stage of life.

Although our data cover a wide time lag (last 40 years) and might therefore reflect past rather than current lack of identification of EDs and related healthcare provision in the UK, it is nevertheless surprising that, across their lifetime, very few women had sought or received treatment for EDs.

Our investigation of risk factors of lifetime EDs revealed important findings. Childhood sexual abuse, unhappiness, and low parental care were associated with binge and/or purge-type ED (AN-BP, full and sub-threshold BN and BED). The association between childhood sexual abuse and binge and/or purge-type ED is consistent with previous retrospective studies [14, 37, 38], and extends this evidence to sub-threshold ED. In line with our recent meta-analysis [11] and previous hypotheses that parenting risk factors and parental influences might act differently across the ED diagnostic spectrum [14], parental overprotection and low maternal care were associated with binge and/or purge disorders, but not AN. We recently showed that retrospectively reported parental influences (including poor parenting and overprotection) predicted body dissatisfaction in women with BN and AN-BP but not AN-R [39]. This association with binge/purge type disorders maybe mediated via negative affect, low self-esteem, or body dissatisfaction developmentally, as might be the case with sexual abuse. Further longitudinal studies are required to empirically test these pathways.

High interpersonal sensitivity was associated with all EDs. Interpersonal sensitivity has been described as sensitivity to other’s feedback and fear of social rejection [26], and it is characterized by misinterpretation of interpersonal behaviors, interpersonal avoidance and discomfort in the presence of others due to a sense of inadequacy. Our finding confirms and strengthens existing cross-sectional evidence that social impairment and interpersonal difficulties are common across EDs [11], and might contribute to their onset and maintenance [11, 40].

We replicated associations identified in clinical studies between high IQ and AN [41, 42], in a community setting (women with lifetime AN-BP had a total IQ on average 5 points higher than women with no EDs). Whether the higher IQ observed is secondary to higher levels of perfectionism, or indeed indexes specific cognitive strengths requires further study and elucidation.

This study is the first to investigate childhood risk factors for PD, a newly described ED. The only twin study of PD recently showed that non-shared environmental factors explained 56% of the variance for PD [43]; however, the study could not disentangle the effect of non-shared environment versus genetic factors. Our findings suggest a role for childhood experiences and parenting as risk factors for PD. Similarly, this is the first study to investigate risk factors for atypical AN, with initial evidence of a risk factor profile more similar to binge/purge type ED than AN-R. This is the first study to show a similar pattern of risk for full threshold and sub-threshold BN and BED. These findings, together with evidence of similar outcomes between threshold and sub-threshold BN and BED [4], confirm similarities between full and sub-threshold ED, in this case in relation to risk factors.

Few associations were identified between environmental risk variables and restrictive AN. This finding might reflect our hierarchical approach to lifetime diagnosis, in that to be included in this group, women had to have met criteria for AN-R only (and not other EDs). As such, our findings point to a smaller contribution of environmental risk to this phenotype [34].

Strengths of the study include a large community sample of women, overcoming bias introduced by studying treatment-seeking individuals. The two-phase epidemiological design, one of the best approaches to estimate prevalence of disease [44], and the survey analytical techniques allowed more accurate estimates to be obtained using our entire Phase I sample. We used a validated and reliable assessment for EDs and supplemented this with a longitudinal assessment of lifetime symptoms to obtain DSM-5 diagnoses. The availability of risk factor data independently collected 20 years prior to the current study allowed a less biased estimation of risk factors, although recall bias might explain some of our findings.

Limitations of the study include the nature of the ALSPAC cohort, i.e. women who were pregnant at a specific point in time in a defined geographic area. The sample is therefore likely to include women with ED who were able to become pregnant at least once and is therefore not representative of the general population. Nevertheless, the lowest ever self-reported BMI in this sample was 10.7, and the lowest measured BMI at mean age 48 years was 15.4, suggesting a range of ED severity within the sample. Participation in Phase 1 was selective; however, we were able to determine that more educated women and those with fewer children participated. Despite attrition between Phase 1 and 2, our analytical approach allows minimizing bias due to attrition. Moreover, risk factor analyses were controlled for socio-demographic factors associated with non-participation in Phase 1, therefore increasing generalizability of the findings. It is possible that women with higher levels of psychopathology were less represented in this study; however, levels of self-reported EDs at enrolment were comparable across participants and non-participants, therefore we are unlikely to have underestimated the prevalence of EDs. Small sample size in some diagnostic groups might account for false negatives. Similarly, chance might explain some of our positive findings. We could not directly investigate other psychiatric disorders and, therefore, the specificity of risk factors for ED versus other psychopathology needs elucidating further.

## Conclusions

EDs are common across the lifespan and in mid-life. Poor healthcare access was evident in this sample of women. This has implications for service provision, which at present is not specifically geared towards women in mid-life, and in identification of women who might be misdiagnosed given the lack of awareness amongst healthcare professionals of ED presentations. Although some risk factors differed across ED subtypes, childhood sexual abuse and poor parenting were associated with binge/purge type disorders, whilst personality factors were more broadly associated with several diagnostic categories. Few risk factors were specifically associated with one diagnostic category. These patterns suggest shared environmental risk across the ED diagnostic spectrum, independent of full/sub-threshold symptoms. The evidence that lifetime and active EDs are common amongst women in mid-life, compounded by the lack of healthcare access and treatment, highlights the likelihood of high disease burden and unmet needs. Future studies should also aim to better characterize EDs in mid-life, and clarify their correlates in terms of physical and psychiatric comorbidities, as well as differences in precipitating factors leading to ‘late onset’ compared to adolescent/young adult onset.

## Additional file

Additional file 1: Table S1. Diagnostic algorithm for DSM-5 ED. **Table S2.** Socio-demographic characteristics of respondents versus non-respondents in Phase 1. **Table S3.** Univariable associations between eating disorder correlates and precursors: OR (95% CI) from logistic regression and mean differences (95% CI) from linear regression. (DOCX 28 kb)

## Abbreviations

ALSPAC: Avon Longitudinal Study of Parents and Children
AN: anorexia nervosa
AN-BP: anorexia nervosa binge-purge
AN-R: anorexia nervosa restrictive
BED: binge eating disorder
BMI: body mass index
BN: bulimia nervosa
DSM-5: Diagnostic and Statistic Manual for Mental Disorders 5^th^ edition
ED: Eating Disorder
EDDS: eating disorders diagnostic schedule
IQ: intelligence quotient
LOC: locus of control
OSFED: other specified feeding and eating disorder
PD: purging disorder
SCID-I: Structured Clinical Interview for DSM-IV-TR disorders

## References

[1] Kessler RC, Berglund PA, Chiu WT, Deitz AC, Hudson JI, Shahly V, Aguilar-Gaxiola S, Alonso J, Angermeyer MC, Benjet C (2013). The prevalence and correlates of binge eating disorder in the World Health Organization World Mental Health Surveys. Biol Psychiatry.

[2] Keshaviah A, Edkins K, Hastings ER, Krishna M, Franko DL, Herzog DB, Thomas JJ, Murray HB, Eddy KT (2014). Re-examining premature mortality in anorexia nervosa: a meta-analysis redux. Compr Psychiatry.

[3] Chesney E, Goodwin GM, Fazel S (2014). Risks of all-cause and suicide mortality in mental disorders: a meta-review. World Psychiatry.

[4] Micali N, Solmi F, Horton NJ, Crosby RD, Eddy KT, Calzo JP, Sonneville KR, Swanson SA, Field AE (2015). Adolescent eating disorders predict psychiatric, high-risk behaviors and weight outcomes in young adulthood. J Am Acad Child Adolesc Psychiatry.

[5] American Psychiatric Association. Diagnostic and statistical manual of mental disorders. 5th ed. Arlington: American Psychiatric Association; 2013.

[6] Smink FR, van Hoeken D, Oldehinkel AJ, Hoek HW (2014). Prevalence and severity of DSM-5 eating disorders in a community cohort of adolescents. Int J Eat Disord.

[7] Stice E, Marti CN, Rohde P (2013). Prevalence, incidence, impairment, and course of the proposed DSM-5 eating disorder diagnoses in an 8-year prospective community study of young women. J Abnorm Psychol.

[8] Solmi F, Hotopf M, Hatch SL, Treasure J, Micali N (2016). Eating disorders in a multi-ethnic inner-city UK sample: prevalence, comorbidity and service use. Soc Psychiatry Psychiatr Epidemiol.

[9] Hay P, Girosi F, Mond J (2015). Prevalence and sociodemographic correlates of DSM-5 eating disorders in the Australian population. J Eat Disord..

[10] Jacobi C, Hayward C, de Zwaan M, Kraemer HC, Agras WS (2004). Coming to terms with risk factors for eating disorders: application of risk terminology and suggestions for a general taxonomy. Psychol Bull.

[11] Caglar-Nazali HP, Corfield F, Cardi V, Ambwani S, Leppanen J, Olabintan O, Deriziotis S, Hadjimichalis A, Scognamiglio P, Eshkevari E (2014). A systematic review and meta-analysis of ‘Systems for Social Processes’ in eating disorders. Neurosci Biobehav Rev..

[12] Micali N, De Stavola B, Ploubidis G, Simonoff E, Treasure J, Field AE (2015). Adolescent eating disorder behaviours and cognitions: gender-specific effects of child, maternal and family risk factors. Br J Psychiatry.

[13] Keel PK, Forney KJ (2013). Psychosocial risk factors for eating disorders. Int J Eat Disord.

[14] Hilbert A, Pike KM, Goldschmidt AB, Wilfley DE, Fairburn CG, Dohm FA, Walsh BT, Striegel WR (2014). Risk factors across the eating disorders. Psychiatry Res.

[15] Boyd A, Golding J, Macleod J, Lawlor DA, Fraser A, Henderson J, Molloy L, Ness A, Ring S, Davey Smith G (2013). Cohort Profile: the ‘children of the 90s’--the index offspring of the Avon Longitudinal Study of Parents and Children. Int J Epidemiol.

[16] Stice E, Telch CF, Rizvi SL (2000). Development and validation of the Eating Disorder Diagnostic Scale: a brief self-report measure of anorexia, bulimia, and binge-eating disorder. Psychol Assess.

[17] First MB, Spitzer RL, Gibbon M, Williams JBW (2002). Structured Clinical Interview for DSM-IV-TR Axis I Disorders, Research Version, Non-Patient Edition (SCID-I/NP).

[18] Keller MB, Lavori PW, Friedman B, Nielsen E, Endicott J, McDonald-Scott P, Andreasen NC (1987). The Longitudinal Interval Follow-up Evaluation. A comprehensive method for assessing outcome in prospective longitudinal studies. Arch Gen Psychiatry.

[19] Anderluh M, Tchanturia K, Rabe-Hesketh S, Collier D, Treasure J (2009). Lifetime course of eating disorders: design and validity testing of a new strategy to define the eating disorders phenotype. Psychol Med.

[20] Brown GW, Harris TO, Peto J (1973). Life events and psychiatric disorders. 2. Nature of causal link. Psychol Med.

[21] Barnett BE, Hanna B, Parker G (1983). Life event scales for obstetric groups. J Psychosom Res.

[22] Dorrington S, Zammit S, Asher L, Evans J, Heron J, Lewis G (2014). Perinatal maternal life events and psychotic experiences in children at twelve years in a birth cohort study. Schizophr Res.

[23] Parker G, Tupling H, Brown LB (1979). Parental bonding instrument. Brit J Med Psychol.

[24] Nowicki S. Adult Nowicki-Strickland internal-external locus of control scale, test manual available from S Nowicki, Jr, Department of Psychology, Emory University, Atlanta, GA. 1976

[25] Boyce P, Parker G (1989). Development of a scale to measure interpersonal sensitivity. Aust N Z J Psychiatry.

[26] Harb GC, Heimberg RG, Fresco DM, Schneier FR, Liebowitz MR (2002). The psychometric properties of the Interpersonal Sensitivity Measure in social anxiety disorder. Behav Res Ther.

[27] Wechsler D (1955). Manual for the Wechsler adult intelligence scale.

[28] Fraser A, Macdonald-Wallis C, Tilling K, Boyd A, Golding J, Davey Smith G, Henderson J, Macleod J, Molloy L, Ness A (2013). Cohort Profile: the Avon Longitudinal Study of Parents and Children: ALSPAC mothers cohort. Int J Epidemiol.

[29] StataCorp (2013). Stata Statistical Software: Release 13.

[30] Dunn G, Pickles A, Tansella M, Vazquez-Barquero JL (1999). Two-phase epidemiological surveys in psychiatric research. Br J Psychiatry..

[31] Pickles A, Dunn G, Vazquez-Barquero JL (1995). Screening for stratification in two-phase (‘two-stage’) epidemiological surveys. Stat Methods Med Res.

[32] Eddy KT, Dorer DJ, Franko DL, Tahilani K, Thompson-Brenner H, Herzog DB (2008). Diagnostic crossover in anorexia nervosa and bulimia nervosa: implications for DSM-V. Am J Psychiatry.

[33] Micali N, Holliday J, Karwautz A, Haidvogl M, Wagner G, Fernandez-Aranda F, Badia A, Gimenez L, Solano R, Brecelj-Anderluh M (2007). Childhood eating and weight in eating disorders: a multi-centre European study of affected women and their unaffected sisters. Psychother Psychosom.

[34] Treasure J, Zipfel S, Micali N, Wade T, Stice E, Claudino A, Schmidt U, Frank GK, Bulik CM, Wentz E (2015). Anorexia nervosa. Nat Rev Dis Primers..

[35] Hudson JI, Hiripi E, Pope HG, Kessler RC (2007). The prevalence and correlates of eating disorders in the National Comorbidity Survey Replication. Biol Psychiatry.

[36] Thomas JJ, Koh KA, Eddy KT, Hartmann AS, Murray HB, Gorman MJ, Sogg S, Becker AE (2014). Do DSM-5 eating disorder criteria overpathologize normative eating patterns among individuals with obesity?. J Obes..

[37] Fairburn CG, Welch SL, Doll HA, Davies BA, O’Connor ME (1997). Risk factors for bulimia nervosa. A community-based case-control study. Arch Gen Psychiatry.

[38] Fairburn CG, Doll HA, Welch SL, Hay PJ, Davies BA, O’Connor ME (1998). Risk factors for binge eating disorder: a community-based, case-control study. Arch Gen Psychiatry.

[39] Krug I, Fuller-Tyszkiewicz M, Anderluh M, Bellodi L, Bagnoli S, Collier D, Fernandez-Aranda F, Karwautz A, Mitchell S, Nacmias B (2015). A new social-family model for eating disorders: A European multicentre project using a case-control design. Appetite..

[40] Treasure J, Schmidt U (2013). The cognitive-interpersonal maintenance model of anorexia nervosa revisited: a summary of the evidence for cognitive, socio-emotional and interpersonal predisposing and perpetuating factors. J Eat Disord..

[41] Lopez C, Stahl D, Tchanturia K (2010). Estimated intelligence quotient in anorexia nervosa: a systematic review and meta-analysis of the literature. Ann Gen Psychiatry..

[42] Kothari R, Solmi F, Treasure J, Micali N (2013). The neuropsychological profile of children at high risk of developing an eating disorder. Psychol Med.

[43] Munn-Chernoff MA, Keel PK, Klump KL, Grant JD, Bucholz KK, Madden PA, Heath AC, Duncan AE (2015). Prevalence of and familial influences on purging disorder in a community sample of female twins. Int J Eat Disord.

[44] Wakefield J, Haneuse SJ (2008). Overcoming ecologic bias using the two-phase study design. Am J Epidemiol.

