# Supplementary material for: Lifetime and 12-month prevalence of eating disorders amongst women in mid-life: a population-based study of diagnoses and risk factors
Source: BMC Med. 2017 Jan 17;15:12. doi: 10.1186/s12916-016-0766-4 (PMC5240354; doi:10.1186/s12916-016-0766-4)
Supplement: Additional file 1: Table S1. — Diagnostic algorithm for DSM-5 ED. Table S2. Socio-demographic characteristics of respondents versus non-respondents in Phase 1. Table S3. Univariable associations between eating disorder correlates and precursors: OR (95% CI) from logistic regression and mean differences (95% CI) from linear regression. (DOCX 28 kb) [file 12916_2016_766_MOESM1_ESM.docx]

**Table S1: Diagnostic algorithm for DSM-5 ED**

| **Eating disorder** |  |  |
| --- | --- | --- |
| **Anorexia**  **Nervosa**  **(AN)** | -Endorses DSM-5 criteria A, B, C and BMI<17.5^1^ |  |
|  | -Endorses at least two criteria (A, B, C), one criterion can be inferred, AND BMI<17.5^1^ | *Criterion A can be inferred by BMI*  *< 17.5; Criterion B can be inferred by restrictive behaviors;*  *Criterion C can be inferred by weight and shape concern* |
| **Bulimia Nervosa (BN)** | -Endorses DSM-5 Criteria |  |
| **Binge-eating Disorder (BED)** | -Endorses DSM-5 Criteria |  |
| **Purging Disorder** | Purging in the absence of binge eating at least once a week for a minimum of 3 months |  |
| **Sub-threshold BN** | -Endorses DSM-5 Criteria for BN but binge eating and purging frequency < once a week or duration<3 months |  |
| **Sub-threshold BED** | -Endorses DSM-5 Criteria for BED but binge eating frequency < once a week or duration<3 months or no distress reported |  |
| **Atypical AN** | -Endorses DSM-5 criteria A, B, C for AN and BMI>18.5 |  |
|  | - Endorses 2 DSM-5 criteria (A, B, C) for AN and BMI>18.5 | *Criterion B can be inferred by restrictive behaviors;* |
| **Other Other Specified Feeding or Eating Disorder** | -Endorses weight and shape concern AND at least one ED behavior (restrictive eating, excessive exercising, purging) AND does not meet criteria for any other ED |  |

BMI (Body Mass Index), ED (Eating Disorder)

1: For women reporting onset of AN prior to age 18, self-reported BMI during AN was adjusted to age-appropriate cut-offs corresponding to a BMI of <17.5 as per Cole TJ, Flegal KM, Nicholls D, Jackson AA. Body mass index cut offs to define thinness in children and adolescents: International survey. *Bmj.* Jul 28 2007; 335(7612): 194.

**Table S2: Socio-demographic characteristics of respondents vs. non-respondents in Phase 1**

|  | N | Respondents (n=5,653) | Non-Respondents  (n=3,576) | Statistic |
| --- | --- | --- | --- | --- |
| Education  Secondary education and above, N (%) | 8,225 | 2,443 (47.3%) | 880 (28.8%) | χ^2^=272.0  p<0.0005 |
| Parity  Multiparae, N (%) | 8,261 | 2,721 (52.9%) | 1,802 (57.9%) | χ^2^=19.81  p<0.0005 |
| Ethnicity  White, N (%) | 8,200 | 5,049 (97.9%) | 2,988 (98.1%) | χ^2^=0.33  p=0.56 |
| Self-reported lifetime ED in pregnancy, N (%) | 8,473 | 193 (3.7%) | 119 (3.7%) | χ^2^=1.26  p=0.7 |
| Age at enrolment, Mean (SD) | 8.471 | 29.4 (4.5) | 27.7 (4.7) | F=285.2,  p<0.001 |
| Age at assessment, Mean (SD) | 5,653 | 47.78 (4.5) | **-** | **-** |

|  | AN-R  (N=30) | AN-BP  (N=41) | BN  (N=55) | BED  (N=61) | Sub-Threshold BN  (N=21) | Sub-Threshold BED  (N=16) | Atypical AN  (N=28) | Other OSFED  (N=18) | PD  (N=27) |
| --- | --- | --- | --- | --- | --- | --- | --- | --- | --- |
| **Risk factors** | **OR (95%CI)** | **OR (95%CI)** | **OR (95%CI)** | **OR (95%CI)** | **OR (95%CI)** | **OR (95%CI)** | **OR (95%CI)** | **OR (95%CI)** | **OR (95%CI)** |
| Adopted or taken into care | (N=27)  - | (N=40)  2.23 (0.62-7.96) | 0.49 (0.1-3.8) | (N=59)  1.43(0.41-5.04) | (N=19)  - | 1.78(0.22-14.39) | (N=27)  1.11(0.14-8.78) | - | (N=26)  1.46 (0.32-6.74) |
| Death of carer | 0.91 (0.25-3.3) | (N=42)  0.95 (0.28-3.23) | 1.23 (0.47-3.29) | 1.60(0.68-3.77) | 1.37(0.30-6.18) | 0.82 (0.10-6.47) | 2.25(0.73-6.88) | 2.13(0.60-7.55) | 6.02**(2.02-17.87) |
| Parental separation or divorce | 2.9 (0.95-8.8) | (N=40)  1.14(0.46-2.82) | 2.00*(1.02-3.91) | 2.10*(1.11-3.97) | (N=20)  - | 1.49(0.41-5.38) | 2.87* (1.20-6.86) | 0.25(0.03-1.99) | 1.19(0.45-3.09) |
| Child sexual abuse (any) | (N=28)  1.73 (0.69-4.31) | 3.67***(1.91-7.04) | (N=54)  4.33***(2.40-7.84) | (N=59)  3.25***(1.86-5.68) | 3.15*(1.27-7.84) | 8.47***(1.86-25.06) | (N=27)  2.05 (0.84-4.99) | (N=17)  3.06(0.78-11.97) | (N=25)  1.72(0.73-4.02) |
| Childhood unhappiness | 2.29 (0.93-5.62) | 1.93 (0.88-4.22) | (N=54)  3.15***(1.68-5.90) | 3.60***(2.00-6.46) | 1.71(0.55-5.29) | 2.28(0.71-7.31) | 2.06 (0.79-5.31) | 2.84 (0.91-8.83) | 2.72*(1.19-6.25) |
| Weighted life event score | (N=29)  1.04 (1.01-1.09) | (N=42)  1.05***(1.03-1.09) | 1.06***(1.04-1.09) | (N=60)  1.08***(1.05-1.10) | (N=22)  1.04*(1.01-1.07) | 1.06*(1.02-1.11) | 1.09***(1.06-1.13) | 1.02(0.95-1.08) | (N=26)  1.04*(1.01-1.08) |
| Parental bonding |  | | | | | | | | |
| -Maternal care  Top quartile  Bottom quartile | 0.80 (0.54-1.19)  1.94 (0.79-4.75) | (N=42)  0.85 (0.71-1.04)  1.59 (0.73-3.44) | (N=55)  0.81*(0.68-0.97)  1.62(0.82-3.21) | 0.92(0.79-1.06)  1.89*(1.01-3.56) | 0.76(0.56-1.05)  1.03(0.29-3.60) | 0.82(0.60-1.13)  7.47***(2.69-20.71) | 1.74(0.67-4.49)  1.09(0.89-1.34) | 1.17 (0.84-1.63)  1.64(0.52-5.19) | 0.96 (0.73-1.27)  3.30**(1.47-7.41) |
| -Parental overprotection | 0.98 (0.90-1.07) | 1.07*(1.01-1.15) | 1.05 (0.98-1.13) | 1.12***(1.04-1.20) | 1.13*(1.04-1.22) | 1.04(0.89-1.21) | 1.11*(1.01-1.22) | 1.10(0.93-1.30) | 1.18***(1.09-1.28) |
| Locus of control | 1.01 (0.92-1.30) | 1.13 (0.96-1.32) | 1.03(0.89-1.19) | 1.14*(0.99-1.33) | 1.20(0.93-1.54) | 1.11(0.87-1.43) | 1.18(0.98-1.43) | 1.09 (0.80-1.48) | 1.11 (0.93-1.33) |
| Interpersonal sensitivity | 1.04* (1.00-1.08) | 1.04***(1.03-1.07) | (N=56)  1.06***(1.04-1.082) | 1.04***(1.02-1.06) | 1.03**(1.01-1.05) | 1.04*(1.00-1.07) | 1.04**(1.02-1.07) | 0.98(0.95-1.01) | (N=26)  1.01 (0.99-1.04) |
| **Fixed factors** |  | | | | | | | | |
| WASI total IQ score^1^ | 1.01 (0.95-1.06) | 1.02 (0.99-1.06) | 1.01(0.98-1.04) | 1.02(0.98-1.06) | 1.00(0.96-1.04) | 1.04(0.97-1.11) | 0.99(0.95-1.04) | 0.97(0.93-1.02) | 1.01(0.98-1.04) |

Table S3: Univariable associations between ED^,^ correlates and precursors: OR (95%CI) from logistic regression and mean differences (95%CI) from linear regression

^1^ available on n= 2165, adjusted for ethnicity and age at assessment;

*:p≤0.05; **p≤0.001; ***:p<0.0001; 4,522 women are the referent group
